# Spectrum of Upper Gastrointestinal Endoscopic Findings in Patients With End-Stage Renal Disease Undergoing Maintenance Hemodialysis: A Single-Center Experience From Pakistan

**DOI:** 10.7759/cureus.112597

**Published:** 2026-07-13

**Authors:** Ghazi Abrar, Salman Ahsam, Nadeem Bajkani, Raja Taha Yaseen, Syed Mudassir Laeeq, Danish Kumar, Abbas Ali Tasneem, Nasir Hassan Luck

**Affiliations:** 1 Hepatogastroenterology, Sindh Institute of Urology and Transplantation, Karachi, PAK; 2 Gastroenterology, Social Security Hospital Faisalabad, Faisalabad, PAK; 3 Gastroenterology and Liver Transplant, Gambat Institute of Medical Sciences, Gambat, PAK; 4 Gastroenterology and Hepatology, Bahrain Specialist Hospital, Manama, BHR; 5 Gastroenterology and Hepatology, Armed Forces Hospital Southern Region, Khamis Mushait, SAU

**Keywords:** end-stage renal disease (esrd), erosive gastropathy, gastritis, maintenance hemodialysis, pakistan, peptic ulcer disease (pud), upper gastrointestinal endoscopy (ogd)

## Abstract

Introduction: Symptoms related to the upper gastrointestinal (GI) tract and bleeding complications are common presentations in patients with end-stage renal disease (ESRD) receiving maintenance hemodialysis. Various factors, including uremia, platelet dysfunction, use of anticoagulation during hemodialysis, anemia, impaired GI motility, diabetes mellitus, and polypharmacy, can lead to a wide range of abnormalities. Local literature from Pakistan is scant. Therefore, the aim was to assess the spectrum of upper GI endoscopic findings in hemodialysis patients with ESRD.

Methods: After approval from the ethical review committee at the Sindh Institute of Urology and Transplantation (SIUT), this prospective, descriptive cross-sectional study was conducted at the Department of Hepatogastroenterology, SIUT, Pakistan, between January 2024 and December 2024. Patients aged 18-65 years suffering from ESRD receiving maintenance hemodialysis for at least three months who had undergone upper GI endoscopy for dyspepsia, vomiting, reflux, anemia, hematemesis, melena, or pre-transplant assessment were included. Data regarding demographics, co-morbidities, dialysis factors, endoscopic indications, lab investigations, drug exposure, and endoscopic findings were collected.

Results: A total of 150 patients participated in the study. The mean age was 47.8 ± 13.6 years, and among them, 92 (61.3%) were males. Diabetes mellitus was found in 64 (42.7%) patients, while hypertension was found in 126 (84%) patients. The most common reason for performing the endoscopy was dyspepsia or epigastric pain in 58 (38.7%) patients, followed by anemia of undetermined cause in 35 (23.3%), and upper GI bleed in 27 (18%) patients, respectively. Endoscopic abnormalities were noted in 126 (84%) patients. The most common finding was gastritis in 76 (50.7%) patients, followed by erosive gastropathy in 42 (28.0%), reflux esophagitis in 31 (20.7%), duodenitis in 28 (18.7%), gastric ulcers in 16 (10.7%), and duodenal ulcers in 11 (7.3%) patients, respectively.

Conclusion: Upper GI lesions are common in patients with ESRD on hemodialysis therapy. The common abnormalities include gastritis, erosive gastropathy, esophagitis, duodenitis, and peptic ulcers. Endoscopic examination needs to be done early in patients with symptoms suggestive of upper GI problems. These findings support consideration of timely upper GI endoscopic evaluation in symptomatic patients with ESRD receiving maintenance hemodialysis.

## Introduction

End-stage renal disease (ESRD) is an important public health issue, causing considerable morbidity, mortality, and economic burden [1]. Those who progress towards kidney failure need to undergo maintenance hemodialysis, peritoneal dialysis, or kidney transplantation [2]. Various gastrointestinal problems have been found in patients with advanced chronic kidney disease (CKD) and ESRD [3]. Previous studies have shown that people on dialysis suffer from more abdominal pain, vomiting, constipation, reflux, and dyspepsia than those without renal insufficiency [4,5]. Another study has indicated that problems like delayed gastric emptying, impaired motility, and overgrowth of bacteria may be responsible for gastrointestinal issues seen in chronic renal failure [6,7].

There might be different reasons that increase the likelihood of upper gastrointestinal mucosal disease in patients with hemodialysis. First of all, uremia might affect the protective nature of the mucosal surface and might result in the development of inflammation [8]. Besides, platelet dysfunction and anticoagulation during dialysis may cause an increased bleeding tendency for otherwise insignificant ulcers [8]. Individuals with ESRD might use several drugs like antiplatelet agents, anticoagulants, phosphate binders, oral iron, potassium binders, and analgesics, including NSAIDs; therefore, these drugs can affect their upper gastrointestinal tolerability or predispose them to upper gastrointestinal bleeding [9].

Many pathological changes can be revealed through upper gastrointestinal endoscopy in ESRD patients, ranging from reflux esophagitis to peptic ulcers and malignancies. The importance of these pathologies lies in the fact that most of them can be effectively treated [10]. Finding erosive gastropathy or peptic ulcer disease might lead to proton pump inhibitor therapy, detection and eradication of *Helicobacter pylori*, stopping mucosa-damaging medications, and close control of antithrombotic medications [11]. In addition, identification of angiodysplasia or vascular ectasia might allow endoscopic treatment that will decrease the rate of repeated bleeding [12]. There were several reviews and observational studies that linked end-stage kidney disease to upper gastrointestinal bleeding, peptic ulcers, erosive gastritis, and vascular lesions [13-16].

Despite the clinical significance of these pathologies, there is a scarcity of local data in the ESRD population. Therefore, the main aim of the study was to evaluate the frequency and spectrum of upper gastrointestinal endoscopic abnormalities in ESRD patients undergoing maintenance hemodialysis in Pakistan.

## Materials and methods

Study design and setting

After approval from the ethical review committee, Sindh Institute of Urology and Transplantation (ERC-SIUT) (approval no. 1146), this prospective, descriptive cross-sectional study design was conducted at the Department of Hepatogastroenterology, SIUT, Pakistan, from 1 January 2024 to 31 December 2024.

All the patients aged between 18 and 65 years and undergoing maintenance hemodialysis with diagnostic upper gastrointestinal endoscopy done for clinical indication were enrolled in the study. Indications for endoscopy included dyspepsia, epigastric pain, persistent nausea/vomiting, symptoms of reflux, hematemesis, melena, anemia of unknown etiology, gastrointestinal blood loss, or pre-transplant evaluation.

Patients having chronic liver disease with portal hypertension, those with existing esophageal or gastric varices, patients with a history of surgery involving the esophagus or stomach, those with a history of active gastrointestinal malignancy before undergoing endoscopy, patients with unstable hemodynamics preventing endoscopic investigation, incomplete records of endoscopy, and non-consenting patients were excluded from the study.

Sample size estimation

Taking an expected frequency of abnormal endoscopic findings among patients with ESRD undergoing maintenance hemodialysis of 70%, with a 95% confidence level and 7.5% margin of error, the required sample size came out to be around 144 patients [16,17]. An increase in sample size to 150 patients was done to overcome the possibility of incomplete data recording and to improve the estimation precision.

Data collection procedure

Data was collected related to the demographic profile, including age, sex, body mass index (BMI), and history of smoking. Data regarding clinical condition involve duration of ESRD, duration of hemodialysis, weekly frequency of hemodialysis, presumed causes of kidney failure, presence of associated diseases like diabetes and hypertension, ischemic heart disease, and history of taking medications, including aspirin, clopidogrel, anticoagulants, nonsteroidal anti-inflammatory drugs (NSAIDs), proton pump inhibitors, oral iron, and phosphate binders. Information regarding presenting symptoms and reasons for undergoing endoscopy was also gathered. Laboratory investigations included hemoglobin, platelets, serum albumin, blood urea nitrogen, serum creatinine, and serum potassium taken in the week prior to endoscopy.

Endoscopic assessment

Upper gastrointestinal endoscopy was performed in a fasting state using a standard video endoscope (Olympus EG-190; Olympus Medical Systems Corp., Tokyo, Japan). In case of hemodynamically stable patients, endoscopy was performed preferably on a non-dialysis day or in a stabilized condition after dialysis. Conscious sedation was used based on cardiopulmonary assessment. Esophagus, gastroesophageal junction, stomach, pylorus, duodenal bulb, and second portion of duodenum were examined systematically. Endoscopy was performed by a gastroenterologist with experience performing more than 1,000 endoscopies, and the findings were recorded.

Endoscopic findings included normal endoscopy, reflux esophagitis, esophageal candidiasis, hiatus hernia, gastritis, erosive gastropathy, gastric ulcer, duodenitis, duodenal ulcer, gastric polyp, vascular ectasia/angiodysplasia, and suspected malignancy. The criteria for diagnosis of gastritis are defined by the presence of any sign, including erythema, edema, friability, or mucosal granularity of the gastric mucosa [18]. Erosive gastropathy is defined by the presence of visible erosions or superficial mucosal breaks [19]. The definition of peptic ulcer disease involves the presence of a mucosal defect with visible depth in the stomach or duodenum [20]. Biopsies were taken when needed, especially when there was malignancy, gastric ulcer, nodular mucosa, or *H. pylori* infection. For gastric biopsy, the Sydney classification system was utilized. For reflux esophagitis, the Los Angeles (LA) grading system was used.

Statistical analysis

IBM SPSS Statistics for Windows, Version 26 (Released 2018; IBM Corp., Armonk, New York, United States) was used for data entry and analysis. Data were presented as mean ± standard deviation for continuous data. For categorical data, frequencies and percentages were used. The overall prevalence rate of abnormal endoscopic findings was reported. Description of results was done according to the endoscopy indication and selected clinical factors. Comparison of categorical variables was done using the chi-square test, while the independent samples t-test was used for continuous variables. A p-value of < 0.05 was considered statistically significant.

## Results

Out of 150 patients enrolled in the study, 92 (61.3%) were males, while 58 (38.7%) were females. The mean age was 47.8±13.6 years, and the mean BMI was 23.9±4.2 kg/m^2^. Hypertension proved to be the most common comorbidity, affecting 126 (84%) patients, followed by diabetes in 64 (42.7%) and ischemic heart disease in 28 (18.7%) patients, respectively. The common causes of ESRD included diabetic nephropathy in 56 (37.3%), hypertensive nephropathy in 43 (28.7%), chronic glomerulonephritis in 18 (12%), obstructive uropathy in eight (5.3%), polycystic kidney disease in five (3.3%), and unknown or multifactorial in 20 (13.3%) patients. The mean duration of hemodialysis was 26±3 months. Most patients (112, 74.7%) were on hemodialysis twice a week, while 38 (25.3%) underwent thrice-weekly hemodialysis (Table 1).

**Table 1:** Baseline characteristics of patients with ESRD on maintenance hemodialysis (n=150) ESRD: end-stage renal disease; BMI: body mass index; NSAID: nonsteroidal anti-inflammatory drugs

Variable		n (%)/mean±SD
Age (years) (mean±SD)		47.8±13.6
Male gender		92 (61.3)
Female gender		58 (38.7)
BMI (kg/m^2^) (mean±SD)		23.9±4.2
Diabetes mellitus		64 (42.7)
Hypertension		126 (84)
Ischemic heart disease		28 (18.7)
Smoking history		31 (20.7)
Median duration of hemodialysis (months)		26±8
Hemodialysis twice weekly		112 (74.7)
Hemodialysis three times weekly		38 (25.3)
Aspirin/clopidogrel use		46 (30.7)
Oral anticoagulant use		12 (8)
NSAID exposure		24 (16)
Proton pump inhibitor use		68 (45.3)
Hemoglobin (g/dL) (mean±SD)		9.2±1.7
Serum albumin <3.5 g/dL		61 (40.7)
Indication for upper gastrointestinal endoscopy	Dyspepsia or epigastric pain	58 (38.7)
	Unexplained anemia	35 (23.3)
	Overt upper gastrointestinal bleeding	27 (18)
	Persistent Nausea or vomiting	18(12)
	Heartburn or reflux-related symptoms	8 (5.3)
	Pre-transplant evaluation	4 (2.7)

The most common indication for upper gastrointestinal endoscopy was dyspepsia or epigastric pain, observed in 58 (38.7%) patients. Unexplained anemia was the second most common indication, observed in 35 (23.3%) (23.3%). Overt upper gastrointestinal bleeding, including hematemesis or melena, was present in 27 (18%) patients. Persistent nausea or vomiting was the indication for endoscopy in 18 (12.0%) patients, reflux symptoms in eight (5.3%), and pre-transplant evaluation in four (2.7%) patients, respectively (Table 1).

The overall frequency of abnormal upper gastrointestinal endoscopic findings was 84% (Table 2). Gastritis was the most common abnormality, identified in 76 50.7%) patients. Erosive gastropathy was observed in 42 patients (28.0%). Reflux esophagitis was identified in 31 (20.7%) patients, hiatal hernia in 14 (9.3%), and duodenitis in 28 (18.7%), while gastric ulcer and duodenal ulcer were identified in 16 (10.7%) and 11 (7.3%) patients, respectively. Overall, peptic ulcer disease was present in 27 patients (18.0%). Less common findings included esophageal candidiasis in six (4%) patients; vascular ectasia or angiodysplasia in five (3.3%) patients; gastric polyps in three (2%) patients; while only one (0.7%) patient had an endoscopic appearance suspicious for gastric malignancy.

**Table 2:** Spectrum of upper gastrointestinal endoscopic findings

Endoscopic finding	Frequency, n (%)
Any abnormal endoscopic finding	126 (84)
Gastritis	76 (50.7)
Erosive gastropathy	42 (28)
Reflux esophagitis	31 (20.7)
Duodenitis	28 (18.7)
Gastric ulcer	16 (10.7)
Duodenal ulcer	11 (7.3)
Hiatal hernia	14 (9.3)
Esophageal candidiasis	6 (4)
Vascular ectasia/angiodysplasia	5 (3.3)
Gastric polyp	3 (2.0)
Suspicious gastric growth	1 (0.7)
Normal endoscopy	24 (14)

Among 58 patients with dyspepsia, gastritis was seen in 34 (58.6%), erosive gastropathy in 15 (25.9%), duodenitis in 12 (20.7%), reflux esophagitis in nine (15.5%), and peptic ulcer disease in seven (12%) patients, respectively. Out of 35 patients undergoing endoscopy for unexplained anemia, gastritis was present in 18 (51.4%) patients, erosive gastropathy in 12 (34.3%), peptic ulcer disease in eight (22.8%), reflux esophagitis in six (17.1%), and duodenitis in five (14.2%) patients, respectively. Among 27 patients presenting with overt upper gastrointestinal bleeding, the most common endoscopic findings were erosive gastropathy in 13 (48.1%) patients, gastric ulcer in seven (25.9%), duodenal ulcer in five (18.5%), severe reflux esophagitis in four (14.8%), and vascular ectasia or angiodysplasia in two (7.4%) patients, respectively. Endoscopic findings by indication are shown in Table 3.

**Table 3:** Endoscopic findings according to indication for endoscopy UGI: upper gastrointestinal

Finding	Dyspepsia/epigastric pain, n=58, N (%)	Unexplained anemia, n=35, N (%)	UGI bleeding, n=27, N (%)	Vomiting/nausea, n=18, N (%)	Reflux symptoms, n=8, N (%)	Pre-transplant, n=4, N (%)
Normal endoscopy	10 (17.2)	4 (11.4)	1 (3.7)	6 (33.3)	2 (25)	1 (4)
Gastritis	34 (58.6)	18 (51.4)	10 (27)	9 (50)	3 (37.5)	2 (50)
Erosive gastropathy	15 (25.9)	12 (34.3)	13 (48.1)	2 (11.1)	0 (0)	0 (0)
Reflux esophagitis	9 (15.5)	6 (17.1)	4 (14.8)	3 (16.7)	7 (88.5)	2(50)
Duodenitis	12 (20.7)	5 (14.2)	6 (22.2)	4 (22.2)	1 (12.5)	0 (0)
Gastric ulcer	4 (6.9)	4 (11.4)	7 (25.9)	1 (5.5)	0 (0)	0 (0)
Duodenal ulcer	3 (5.1)	4 (11.4)	5 (18.5)	0 (0)	0 (0)	0 (0)
Vascular ectasia/angiodysplasia	0 (0)	3 (8.6)	2 (7.4)	0 (0)	0 (0)	0 (0)
Esophageal candidiasis	1 (1.7)	1 (2.9)	1 (4.2)	2 (11.1)	1 (12.5)	0 (0)

Comparative analysis showed that male sex (p=0.004), comorbid diabetes (p<0.001) and hypertension (p<0.001), longer duration of hemodialysis (p=0.003), use of antiplatelet therapy (p=0.021) or NSAIDs (p=0.013), and lower hemoglobin and serum albumin levels (both p<0.001) were significantly associated with abnormal endoscopic findings in patients with ESRD (Table 4).

**Table 4:** Comparative analysis of different variables in terms of abnormal endoscopic findings NSAID: nonsteroidal anti-inflammatory drugs

Variable	Abnormal endoscopy (n=126), N (%)	Normal endoscopy (n=24), N (%)	p-value
Mean age (years)	49.1±13.2	41.0±14.1	0.054
Male gender	79 (62.7)	13 (54.2)	0.004
Diabetes mellitus	58 (46)	6 (25)	<0.001
Hypertension	108 (85.7)	18 (75)	<0.001
Dialysis duration >2 years	66 (52.4)	9 (37.5)	0.003
Aspirin/clopidogrel use	42 (33.3)	4 (16.7)	0.021
NSAID exposure	23 (18.3)	1 (4.2)	0.013
Hemoglobin <9 g/dL	58 (46)	6 (25)	<0.001
Serum albumin <3.5 g/dL	56 (44.4)	5 (20.8)	<0.001

## Discussion

In this study, upper gastrointestinal endoscopic abnormalities were observed in a large proportion of patients with ESRD receiving maintenance hemodialysis. Overall, 84% of the study population had at least one abnormal endoscopic finding, suggesting that clinically significant upper gastrointestinal mucosal pathology is highly prevalent in this population. The most common abnormalities were gastritis, erosive gastropathy, reflux esophagitis, duodenitis, and peptic ulcer disease. These findings support the concept that patients with ESRD on hemodialysis represent a high-risk population for upper gastrointestinal symptoms, mucosal injury, and bleeding-related pathology.

The high frequency of abnormal endoscopic findings in our study is consistent with previously published literature. Sotoudehmanesh et al. evaluated endoscopic findings in patients with ESRD and reported a wide range of abnormalities, including antral erythema, gastric erosions, duodenal erosions, and duodenal ulcers [10]. Similarly, Usta et al. assessed upper gastrointestinal endoscopic and pathological findings in dialysis patients being evaluated for renal transplantation and found that upper gastrointestinal abnormalities were common even in patients without severe overt symptoms [13]. Gastritis was the most frequent lesion in the present study, affecting 50.7% of patients. This is most likely related to the uremia that may alter gastric mucosal defense mechanisms, impair epithelial repair, and promote mucosal inflammation. In addition, ESRD patients are commonly exposed to multiple medications, including oral iron, phosphate binders, antiplatelet agents, anticoagulants, and NSAIDs, all of which may contribute to dyspeptic symptoms or mucosal injury. Previous studies have also reported inflammatory and erosive gastric lesions as dominant endoscopic findings in dialysis populations [10,13]. The high frequency of gastritis in our cohort may also reflect the burden of *H. pylori* infection in South Asian populations, although routine biopsy-based confirmation was not uniformly available in the present study. This remains an important area for further research because *H. pylori* eradication may reduce ulcer recurrence and improve dyspeptic symptoms in selected patients [11].

Erosive gastropathy was the second most common abnormality in our study, observed in 28% of patients. This finding is clinically important because erosive lesions may contribute to chronic occult blood loss, worsening anemia, and overt upper gastrointestinal bleeding. In patients with ESRD, the bleeding risk is amplified by uremia-associated platelet dysfunction, heparin exposure during dialysis, and frequent antiplatelet or anticoagulant use. Kaur et al. highlighted the close link between kidney disease, cardiovascular comorbidity, antithrombotic exposure, and gastrointestinal bleeding risk in ESRD patients [9]. In our study, erosive gastropathy was particularly common among patients undergoing endoscopy for overt upper gastrointestinal bleeding, where it was identified in 48.1% of cases. This supports the practical value of early endoscopic evaluation in hemodialysis patients presenting with hematemesis, melena, or unexplained anemia.

Peptic ulcer disease was present in 18% of our patients, including gastric ulcers in 10.7% and duodenal ulcers in 7.3%. These results are comparable with previous reports showing that peptic ulcer disease is more common in patients with advanced CKD and ESRD than in the general population [14,15]. The mechanisms are likely multifactorial and include mucosal ischemia, impaired mucosal defense, drug exposure, *H. pylori* infection, and systemic inflammation. In patients with overt bleeding, gastric and duodenal ulcers were detected in 25.9% and 18.5%, respectively, highlighting their importance as bleeding sources in this high-risk population. Marinescu et al. also reported peptic ulcer disease and erosive lesions as important causes of upper gastrointestinal bleeding in CKD patients [15]. Similarly, Laeeq et al., in a Pakistani cohort of ESRD patients with upper gastrointestinal bleeding, found that ulcer disease and erosive mucosal lesions were important endoscopic findings and that some patients required endoscopic therapeutic intervention [16].

Reflux esophagitis was found in 20.7% of our patients, while hiatal hernia was identified in 9.3%. Gastroesophageal reflux symptoms are frequently reported in dialysis patients and may be related to delayed gastric emptying, altered gastrointestinal motility, dietary restrictions, fluid shifts, and autonomic dysfunction, particularly in diabetic patients. Cano et al. reported that gastrointestinal symptoms, including reflux-related symptoms, are common among patients receiving dialysis [4]. Strid et al. also demonstrated that patients with chronic renal failure have increased gastrointestinal symptoms and abnormal intestinal motility, which may contribute to upper gastrointestinal complaints [6,7]. The presence of reflux esophagitis in one-fifth of our cohort emphasizes that esophageal pathology should not be overlooked in ESRD patients presenting with dyspepsia, heartburn, nausea, or anemia.

Vascular ectasia or angiodysplasia was found in 3.3% of the total cohort but was more frequent among patients evaluated for unexplained anemia or overt bleeding. Although less common than inflammatory and ulcerative lesions, vascular lesions are clinically important because they may cause recurrent or persistent bleeding in ESRD patients [21]. Previous literature has shown that CKD is associated with increased risk of gastrointestinal bleeding, including bleeding from vascular lesions [14]. Endoscopic detection is therefore important because angiodysplasia and vascular ectasia may be amenable to endoscopic therapy, including argon plasma coagulation or other modalities [12].

In our study, abnormal endoscopic findings were significantly associated with male gender, diabetes mellitus, hypertension, dialysis duration greater than two years, antiplatelet use, NSAID exposure, hemoglobin below 9 g/dL, and serum albumin below 3.5 g/dL. These associations are clinically meaningful. Diabetes may contribute through autonomic neuropathy, gastroparesis, impaired mucosal healing, and vascular disease [22]. Longer dialysis duration may indicate prolonged exposure to uremia, heparin, inflammation, and medication burden [23]. NSAID and antiplatelet exposure are well-established contributors to mucosal injury and bleeding [24]. Hypoalbuminemia may reflect malnutrition, inflammation, or poor overall health status, all of which may impair mucosal integrity and healing [25]. These findings suggest that risk stratification may help identify hemodialysis patients who require earlier endoscopic evaluation.

This study has certain limitations. It was a single-center study and included only patients who underwent endoscopy for clinical indications; therefore, the results may not be generalizable to all asymptomatic ESRD patients. Secondly, not all patients underwent histopathology and *H. pylori* testing, limiting the ability to correlate endoscopic gastritis with biopsy-proven inflammation or infection. Third, the cross-sectional study design limits the ability to establish the temporal correlation between the potential risk factors and endoscopic findings in patients with ESRD. The study was conducted at SIUT, a specialized tertiary referral center for nephrology, dialysis, and transplantation. Patients treated at such a center may have greater disease severity, more comorbidities, more persistent gastrointestinal symptoms, or a higher likelihood of being referred for endoscopy than patients managed in community or general hospitals. This referral and selection pattern may have contributed to the high frequency of abnormal endoscopic findings observed in the study and may limit the generalizability of the 84% prevalence estimate to the broader maintenance-hemodialysis population. Despite these limitations, the study provides useful local data from Pakistan and highlights the high burden of upper gastrointestinal pathology among patients with ESRD receiving maintenance hemodialysis.

## Conclusions

Upper gastrointestinal endoscopic abnormalities were common among patients with ESRD receiving maintenance hemodialysis. The findings support the importance of considering timely endoscopic evaluation in symptomatic patients, particularly those presenting with dyspepsia, unexplained anemia, or overt upper gastrointestinal bleeding. Larger multicenter studies with routine histopathology, *H. pylori* testing, and multivariable risk assessment are needed to evaluate and identify the predictors of significant upper gastrointestinal lesions in this high-risk population.
